# The association between inflammation, arterial stiffness, oxidized LDL and cardiovascular disease in Finnish men with metabolic syndrome – a 15-year follow-up study

**DOI:** 10.1186/s12872-024-03818-x

**Published:** 2024-03-15

**Authors:** Harri Juhani Saarinen, Jorma Lahtela, Päivi Mähönen, Ari Palomäki, for the Hämeenlinna Metabolic Syndrome Research Program Study Group, Hanna Pohjantähti-Maaroos, Sari Husgafvel, Timo Knuth, Ruth Laitinen, Kalevi Oksanen, Kai Vesterinen, Marja Wallenius

**Affiliations:** 1 Tampere Heart Hospital, Ensitie 4, Tampere, FI-33520 Finland; 2 Tampere University Central Hospital, Teiskontie 35, Tampere, FI-33521 Finland; 3 Vita Laboratories, Laivakatu 5 F, Helsinki, FI-00150 Finland; 4 Department of Bacteriology & Immunology, University of Helsinki, Yliopistonkatu 4, Helsinki, FI-00100 Finland; 5 Department of Emergency Medicine, Kanta–Häme Central Hospital, Ahvenistontie 20, Hämeenlinna, FI-13530 Finland; 6 Cardiometabolic Unit, Linnan Klinikka, Raatihuoneenkatu 10, Hämeenlinna, FI-13100 Finland; 7 Faculty of Medicine and Health Technology, Tampere University, Tampereen Yliopisto, FI-33014 Finland

**Keywords:** Metabolic syndrome, hs-CRP, OxLDL, Arterial stiffness, Cardiovascular disease, Risk calculators, Outcomes, Long-term follow-up

## Abstract

**Background:**

All-cause mortality and cardiovascular disease are increased in subjects with metabolic syndrome (MetS). Risk scores are used to predict individual risk of heart disease. We performed a long-term follow-up study to investigate whether risk scores and cardiovascular risk factors such as arterial stiffness, high-sensitive C-reactive protein (hs-CRP) and oxidized LDL (OxLDL) can be used to predict cardiovascular events in Finnish men with MetS.

**Methods:**

After baseline measurements we followed 105 Finnish men aged 30 to 65 years with MetS for a mean period of 16.4 years. The primary outcome of the study was a composite of myocardial infarction, stroke, symptomatic vascular disease diagnosed with invasive angiography, coronary or peripheral revascularization, amputation due to peripheral vascular disease, cardiovascular death and non-cardiovascular death. The endpoints were retrieved from electronic medical records.

**Results:**

The number of acute myocardial infarctions and strokes during the first 10 years was lower than estimated by FINRISK score but SCORE predicted cardiovascular death correctly. During the whole follow-up period, 27 of 105 participants (25.8%) had 30 endpoint events. The incidence of the primary composite outcome was significantly lower in subjects with hs-CRP < 1.0 mg/L than in subjects with hs-CRP ≥ 1.0 mg/L (6 of 41 subjects [14.6%] vs. 21 of 64 subjects [32.8%]; *p* = 0.036). The incidence of the primary composite outcome was higher among subjects with large artery elasticity classified as borderline compared to subjects with normal large artery elasticity (5 of 10 subjects [50%] vs. 22 of 93 subjects [24%]; *p* = 0.05). There was no difference in the incidence of primary composite outcome in groups with different degrees of small artery elasticity or different level of oxLDL.

**Conclusions:**

Men with MetS who had hs-CRP ≥ 1.0 mg/L had higher risk for CVD and all-cause mortality than those with hs-CRP of < 1.0 mg/L. This also applies to subjects with borderline decreased large artery elasticity. The amount of OxLDL had no predictive value on the incidence of CVD and all-cause mortality. Men with MetS participating in the Hämeenlinna Metabolic Syndrome Research Program without lifestyle or drug intervention had better outcome for myocardial infarction or stroke than estimated by the FINRISK score.

**Trial registration:**

ClinicalTrials.gov NCT01119404 retrospectively registered 07/05/2010.

## Background

Metabolic syndrome (MetS) is defined as a combination of cardiovascular risk factors such as impaired glucose metabolism, elevated blood pressure, central/visceral obesity and dyslipidemia. All-cause and cardiovascular disease (CVD) mortality are increased in men with MetS even in the absence of baseline CVD [1]. The global increase of obesity is likely to increase the prevalence of MetS as well. Thus, more subjects are at risk to develop clinical CVD.

The progression of CVD can be asymptomatic for a long period of time and sometimes the first symptom can be an acute myocardial infarction (AMI) leading to sudden cardiac death (SCD). Primary prevention of CVD and early identification of patients at risk of AMI and SCD is of utmost importance.

This has led to the development of risk calculators such as SCORE [2] and FINRISK [3]. There is also interest in assessing other than the traditional CVD risk factors to estimate the risk of CVD and mortality. High-sensitivity C-reactive protein (hs-CRP) has been shown to associate positively with the risk of CVD events [4]. Central arterial stiffness measured using carotid-femoral pulse wave velocity is an independent predictor of CVD morbidity and mortality [5]. It has been suggested that arterial stiffness could be a useful indicator of aortic atherosclerosis [6]. Oxidized low-density lipoprotein (OxLDL) contributes to the foam cell formation in atherosclerosis and is regarded as more atherogenic than native low-density lipoprotein (LDL) [7].

The aim of this study was to investigate whether unconventional risk factors such as hs-CRP together with arterial elasticity, OxLDL and risk calculator stratification can predict CVD events among Finnish middle-aged men with MetS during a 15-year follow-up.

## Methods

### Study population and follow-up

This study is a long-term register study of the Hämeenlinna Metabolic Syndrome Research Program. The study subjects have been described earlier [8]. In short, the study subjects were 120 men with MetS according to the National Cholesterol Education Program (NCEP) Adult Treatment Panel III criteria [9] aged from 30 to 65 years from the Hämeenlinna region (population 170 000). The study subjects were referred from private and public consultations in primary and secondary health care. In the initial study, in addition to the traditional baseline characteristics, we measured the subjects’ arterial elasticity, hs-CRP and OxLDL concentrations and calculated the 10-year risk of CVD events and mortality using SCORE and FINRISK score models. The FINRISK model uses the subject’s age, sex, smoking status, systolic blood pressure, total cholesterol, high-density lipoprotein cholesterol (HDL-C), diabetes status and possible AMI or stroke of subject’s parents to predict the risk of AMI or stroke during the next 10 years [3]. The SCORE risk model uses the subject’s age, sex, smoking status, systolic blood pressure, total cholesterol or total cholesterol/HDL-C ratio and population-based risk to predict the risk of fatal cardiovascular event during the next 10 years [2].

The incidence of composite endpoints was assessed in three subject groups for both risk score models: low, medium and high risk. For FINRISK, the 10-year risk for fatal or non-fatal AMI or stroke was < 5% in low-risk group, 5-14.9% in medium-risk group and ≥ 15% in high-risk group. For SCORE, the risk of CVD death for the groups were < 3%, 3-4.9% and ≥ 5%, respectively.

The subjects filled a structured questionnaire including their smoking status and typical weekly amount, type and mode of physical activity at baseline. The information on the subjects’ cardiovascular disease status and mortality was retrieved from the region’s electronic medical record (EMR) on 25. March, 2021.

The study followed the ethical principles of the Declaration of Helsinki and each study subject gave a written informed consent. The original study protocol was approved by the Research Ethics Committee of Kanta-Häme Hospital District. The subject recruitment and baseline characteristics were performed 15.6.2004–10.11.2005 so the minimum follow-up data of 15 years was available from all subjects. The follow-up data of ≥ 15 years was collected in a registry setting and the follow-up study was approved by the Ethics Committee of Tampere University Hospital District (no. R19057).

### Blood sampling

Blood samples were drawn after a 12-hour overnight fast with a minimum of 10-minute rest. The serum total cholesterol, low-density lipoprotein cholesterol (LDL-C), HDL-C, triglyserides, OxLDL and glycated hemoglobin (HbA1c) were measured and analyzed in hospital laboratory, which practices strict internal quality control and national external quality assurance program (Labquality Oy, Helsinki, Finland) with daily and monthly control sampling. Cutoffs for OxLDL were following: <60 U/L for low risk, ≥ 60 and < 70 U/L for moderate risk and ≥ 70 U/L for high risk. These robust cutoffs for general guidance are provided by commercial laboratories and they are based on a study of 1,889 apparently healthy individuals without MetS and a reference range study using specimens from 704 apparently healthy volunteers [10].

### Measuring of high-sensitivity CRP

The plasma concentration of hs-CRP test was analyzed according to the validated and accredited method in the Vita Laboratories, Helsinki, Finland. In brief, the analysis of hs-CRP was carried out with a commercially available Bekman Coulter CRP latex reagents used with an Olympus AU620 analyzer (Beckman Coulter, Brea, CA, USA). The measurement range of this immunoturbidometric test was hs-CRP level between 0.2 and 160 mg/l, and the reference value was used as a CRP level 3 mg/l. A hs-CRP-level under 1.0 mg/l indicates a low cardiovascular risk, a hs-CRP-level 1.0–3.0 mg/l indicates a moderate cardiovascular risk, and a hs-CRP-level above 3.0 mg/l indicates a high cardiovascular risk [11]. After assay, the hs-CRP results were validated and released through controlled validation process.

### Measuring of arterial elasticity

The arterial elasticity was measured after a minimum of 10-minute rest with a non-invasive arterial tonometer (HDI/PulseWave™ CR-2000, Hypertension Diagnostics, Eagan, MN, USA) which records the radial artery pulse wave. The tonometer utilizes a modified Windkessel method [12] to report two variables as a mean of five most even pulse waves measured during the recording: capacitive elasticity of large arteries (C1) and the reflective elasticity of small arteries (C2). The C1 depicts the elastic properties of large arteries such as aorta or coronary arteries and the C2 depicts the endothelial function of the microvascular circulation [13]. The measuring technique has been shown to correlate with invasive measures of arterial elasticity with high reproducibility [14, 15]. The C1 and C2 were measured four times for each subject to obtain mean large and small arterial elasticity results. The subjects’ arterial elasticity was graded according to the CR-2000 normal values provided by the manufacturer (Table 1).

**Table 1:** Reference values of arterial elasticity for PulseWave CR-2000

	**C1 – Large arteries**			**C2 – Small arteries**		
Age	Abnormal	Borderline	Normal	Abnormal	Borderline	Normal
** 30–39**	< 8	8–14	> 14	< 6	6–8	> 8
** 40–49**	< 7	7–12	> 12	< 5	5–7	> 7
** 50–59**	< 6	6–11	> 11	< 5	5–7	> 7
** 60–69**	< 5	5–10	> 10	< 4	4–6	> 6

### Outcome

The primary outcome of the study was a composite of (I) the first occurrence of type 1 AMI or cerebral stroke, (II) diagnosis of symptomatic coronary or peripheral artery disease (PAD) diagnosed with invasive angiography, (III) the first occurrence of coronary or peripheral revascularization, (IV) amputation due to PAD, (V) cardiovascular death and (VI) non-cardiovascular death. Only one endpoint event was accepted for each hospitalization i.e., a subject with AMI leading to coronary angiography and percutaneous coronary intervention (PCI) would only be registered as having one endpoint instead of three. Transient ischemic attack (TIA) was not accepted as an endpoint since the diagnosis relies strongly on clinical suspicion in the absence of other plausible causes. A single cardiologist (HS) individually assessed all endpoints and was blinded to the baseline characteristics of the subjects.

### Statistical analysis

Statistical analysis was done with IBM SPSS Statistics (Version: 29.0.1.0, 2023). Cumulative survival curves were generated with the use of the Kaplan-Meier method and difference of survival between groups was tested with the log rank test. The *p*-value of < 0.05 was held statistically significant.

## Results

### Participants

**Fig. 1:**
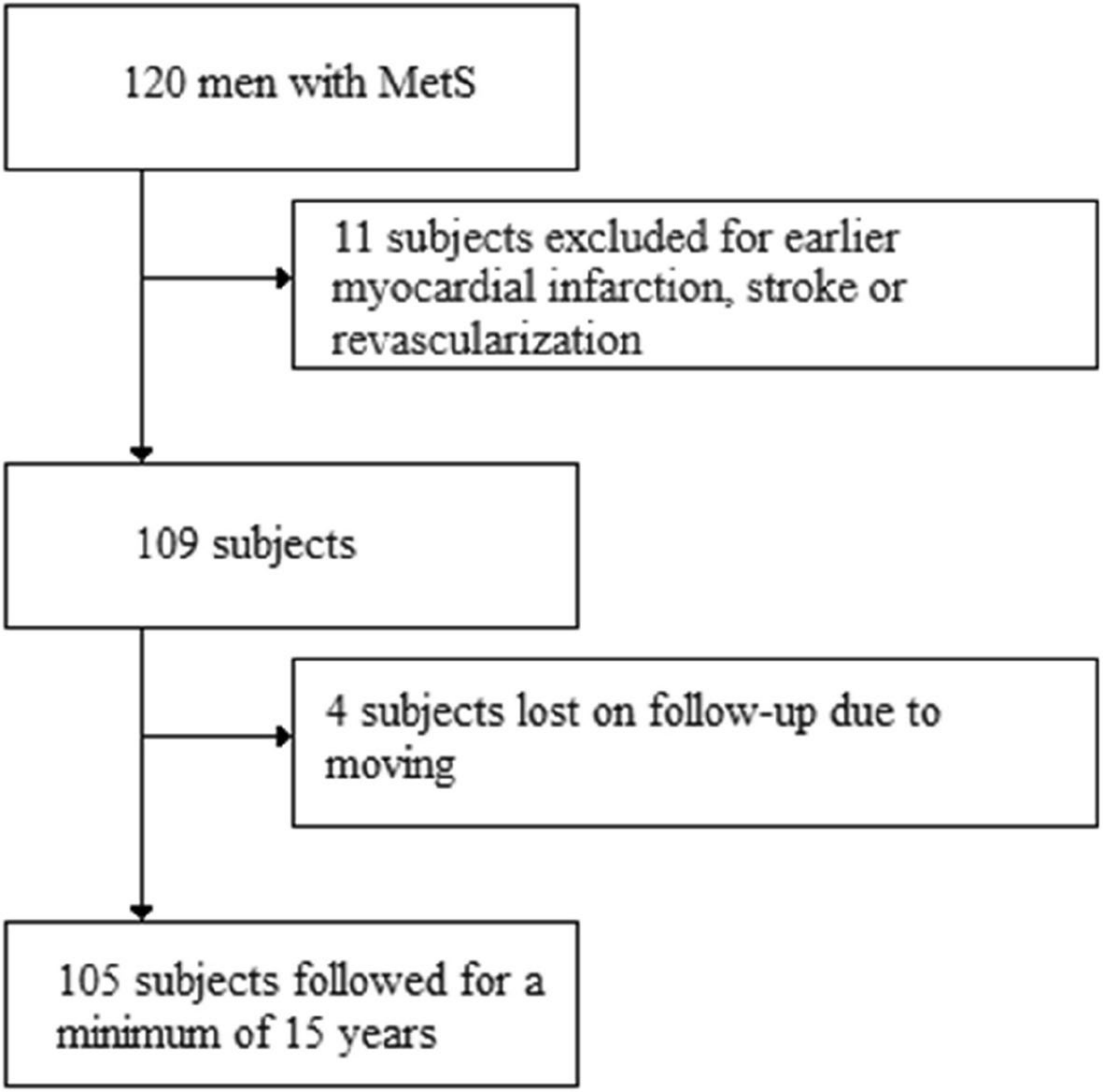
The study flowchart

Since the aim of this study was to predict the possible major cardiovascular outcomes in primary prevention, we excluded 11 subjects with a history of myocardial infarction, coronary revascularization or cerebral stroke. Four subjects were lost to follow-up due to moving to another country or hospital district. We followed 105 men with MetS for a minimum of 15 years and included them in the final analysis (Fig. 1). The mean follow-up period was 16.4 ± 0.3 years. Two subjects did not undergo any arterial elasticity measurements and three subjects did not undergo OxLDL measurements so leaving 103 subjects for the arterial elasticity and 102 subjects for the OxLDL analysis. The demographics of the subjects at baseline are presented in Table 2.

**Table 2:** Baseline characteristics of study population (*n* = 105)

Age, years	50.6 ± 8.4
Waist circumference, cm	113 ± 12
Body mass index, kg/m^2^	31.8 ± 4.9
Systolic blood pressure, mmHg	139 ± 14
Diastolic blood pressure, mmHg	82 ± 8
Resting heart rate, bpm	65 ± 10
Physical activity, kcal/week	1066 ± 1370
Medication	
• Aspirin, n (%)	17 (16%)
• Beta-blockers, n (%)	30 (29%)
• ACE-inhibitors, n (%)	15 (14%)
• ARBs, n (%)	25 (24%)
• CCBs, n (%)	3 (3%)
• Diuretics, n (%)	18 (17%)
• Statins, n (%)	24 (23%)
• Glucose-lowering drugs, n (%)	15 (14%)
Smoking	
• Current, n (%)	21 (20%)
• Former, n (%)	49 (47%)
• Never, n (%)	35 (33%)
Number of pack-years in smokers	16 ± 14
Fasting glucose, mmol/L	6.8 ± 1.8
HbA1c, mmol/L	44.5 ± 11.0
Total cholesterol, mmol/L	5.6 ± 1.4
HDL-Cholesterol, mmol/L	1.2 ± 0.3
LDL-Cholesterol, mmol/L	3.4 ± 1.1
Triglycerides, mmol/L	2.9 ± 3.4
hs-CRP, mg/L	
• Optimal (< 1.0 mg/L)	41 (39%)
• Not optimal (≥ 1.0 mg/L)	64 (61%)
C1, mL/mmHg x 10	
• Normal	93 (90%)
• Borderline decreased	10 (10%)
C2, mL/mmHg x 100	
• Normal	42 (41%)
• Borderline decreased	30 (29%)
• Abnormal	31 (30%)
OxLDL, U/L	
• Low risk (< 60 U/L)	34 (33%)
• Moderate risk (≥ 60 and < 70 U/L)	8 (8%)
• High risk (≥ 70 U/L)	60 (59%)
FINRISK, %	
• Low risk (< 5%)	33 (31%)
• Medium risk (5-14.9%)	45 (43%)
• High risk (≥ 15%)	27 (26%)
SCORE, %	
• Low risk (< 3%)	56 (53%)
• Medium risk (3-4.9%)	25 (24%)
• High risk (≥ 5%)	24 (23%)

The values are presented as number and percentage (%) or mean ± standard deviation

*ACE* Angiotensin-converting enzyme, *ARB* Angiotensin II receptor blocker, *CCB* Calcium channel blocker, *SBP* HbA1c Glycated hemoglobin, *hs-CRP* High-sensitive C-reactive protein, *C1* Capacitive elasticity of large arteries, *C2* Reflective elasticity of small arteries, *OxLDL* Oxidized low-density lipoprotein

**Fig. 2:**
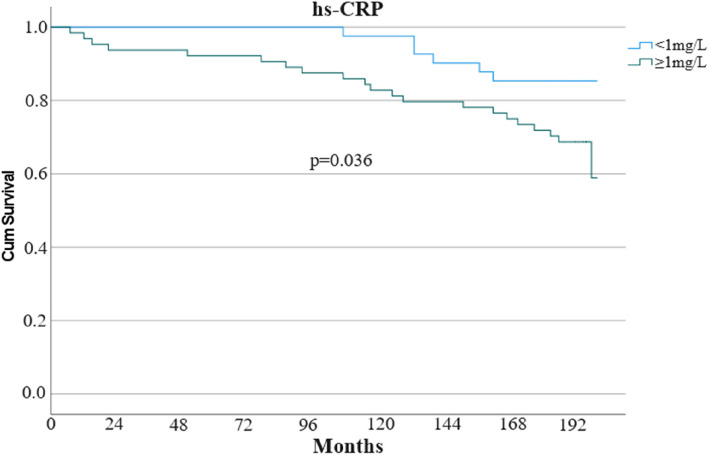
Kaplan-Meier survival curve for groups with low (< 1 mg/L) or not optimal (≥ 1 mg/L) hs-CRP. hs-CRP: High-sensitive C-reactive protein

During the follow-up, 27 of 105 participants (25.8%) had 30 endpoint events. The observed endpoints were non-cardiovascular death (9), cardiovascular death (8), diagnosis of symptomatic vascular disease with invasive angiography (5), cerebral stroke (4), AMI (3) and limb amputation due to PAD (1). The incidence of the primary composite outcome was significantly lower in subjects with hs-CRP < 1.0 mg/L than in subjects with hs-CRP ≥ 1.0 mg/L (6 of 41 subjects [14.6%] vs. 21 of 64 subjects [32.8%]; *p* = 0.036) (Fig. 2).

**Fig. 3:**
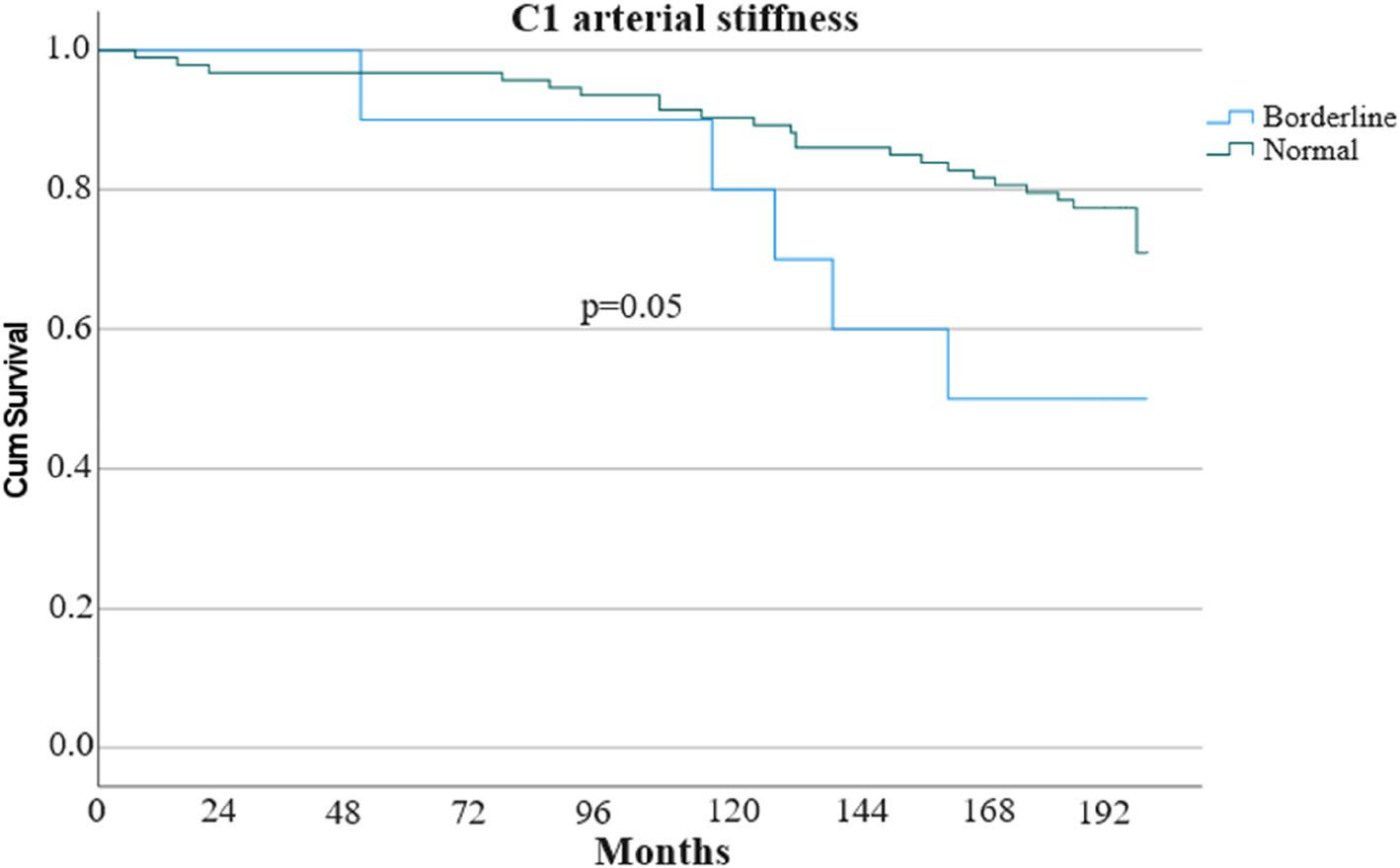
Kaplan-Meier survival curve for groups with borderline decreased and normal large artery elasticity. C1: Capacitive elasticity of large arteries

None of the subjects had obvious impairment of large artery elasticity (C1). However, the incidence of the primary composite outcome was higher among subjects with borderline decreased C1 than among subjects with normal C1 (5 of 10 subjects [50%] vs. 22 of 93 subjects [24%]; *p* = 0.05) (Fig. 3).

**Fig. 4:**
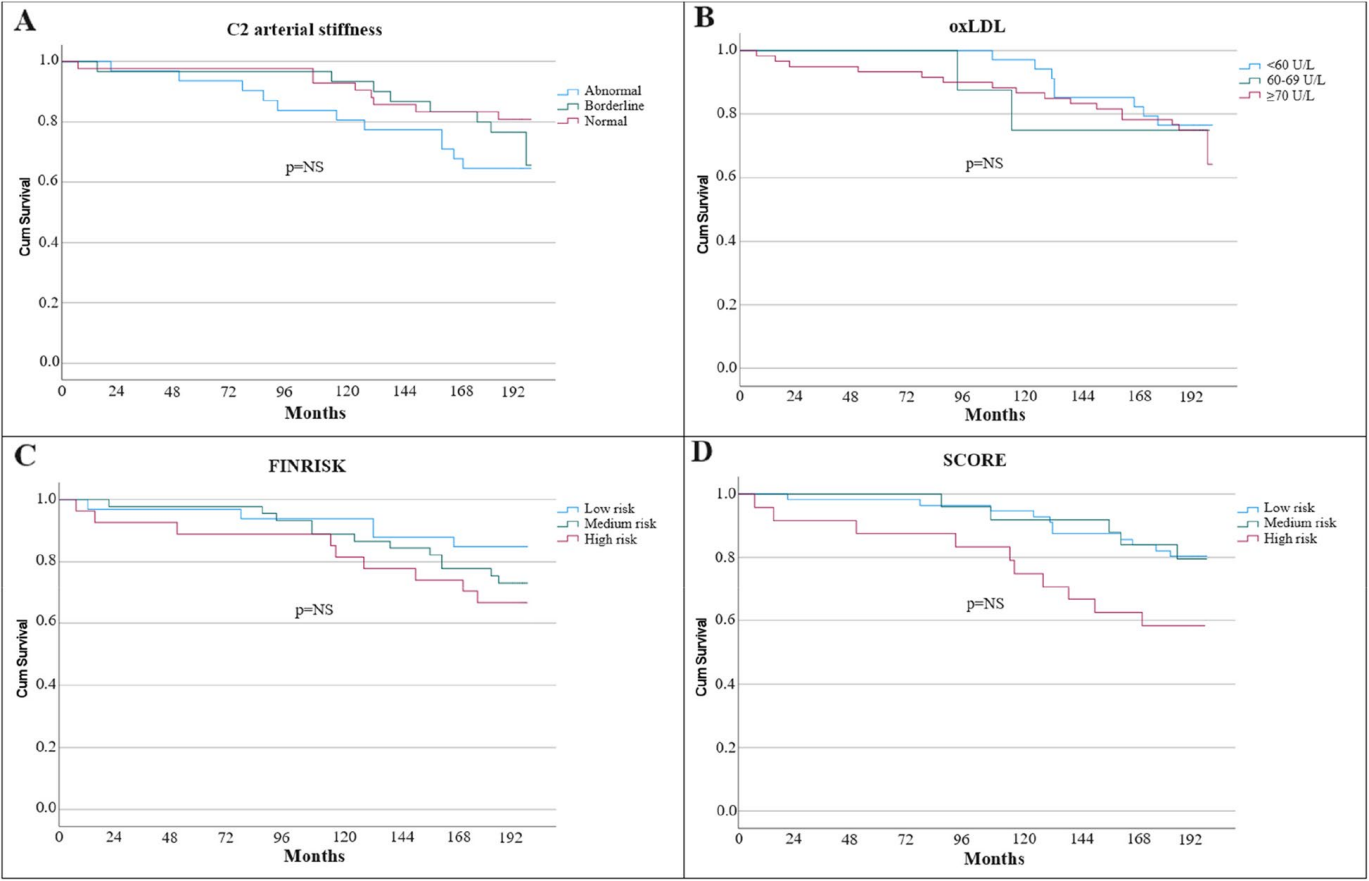
**A**-**D **Kaplan-Meier survival curves for groups with different C2, oxLDL levels and FINRISK/SCORE risk scores. C2: reflective elasticity of small arteries, OxLDL: oxidized low-density lipoprotein

There was no difference in the incidence of primary composite outcome in groups with different degrees of small artery elasticity (Fig. 4A), in groups with different concentrations of OxLDL (Fig. 4B) or in low-, medium- or high-risk subjects according to FINRISK (Fig. 4C) and SCORE models (Fig. 4D).

**Fig. 5:**
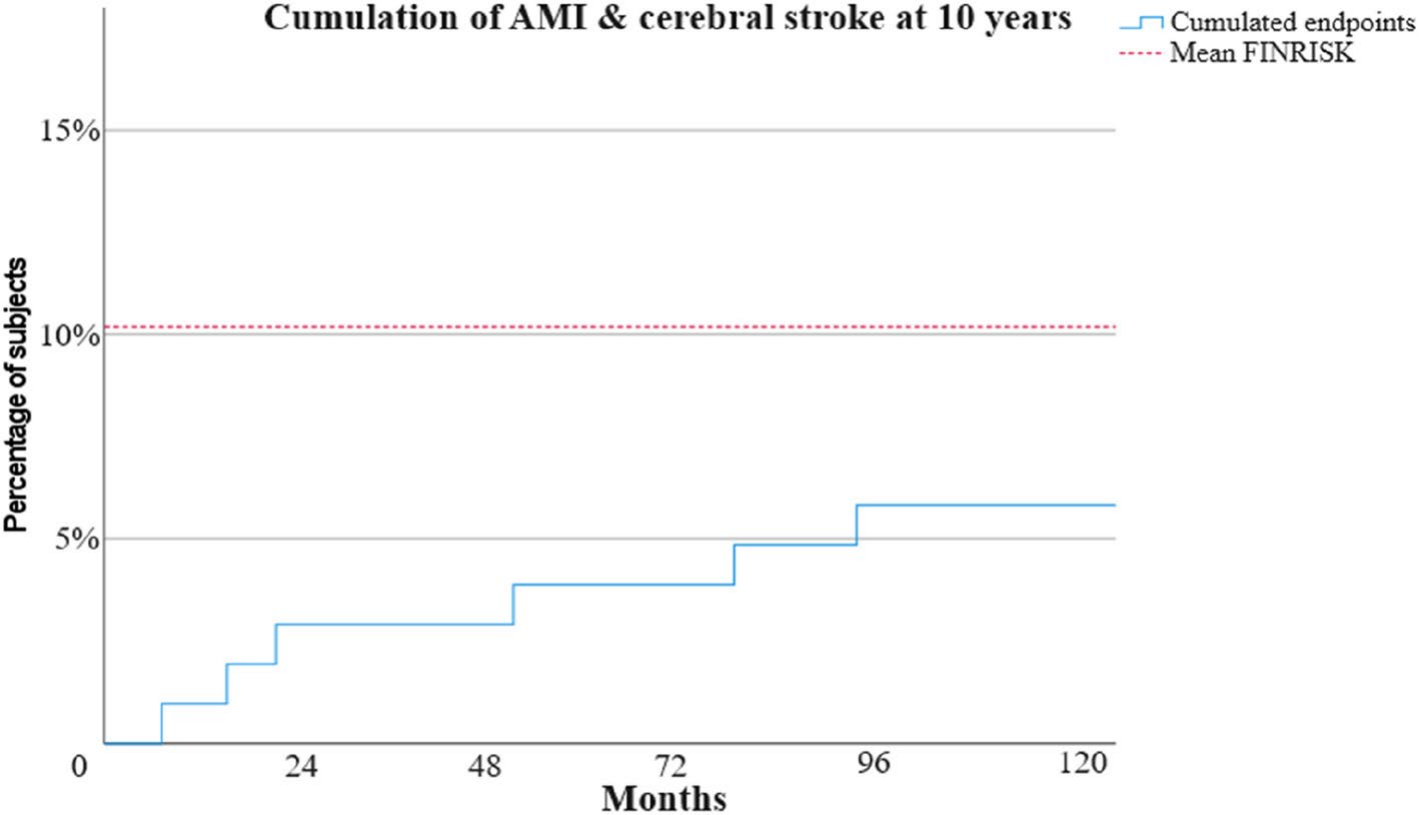
The cumulation of fatal or non-fatal AMI and cerebral stroke during 10 years of follow-up. The dotted red line indicates the mean FINRISK calculated at baseline (10.2%)

**Fig. 6:**
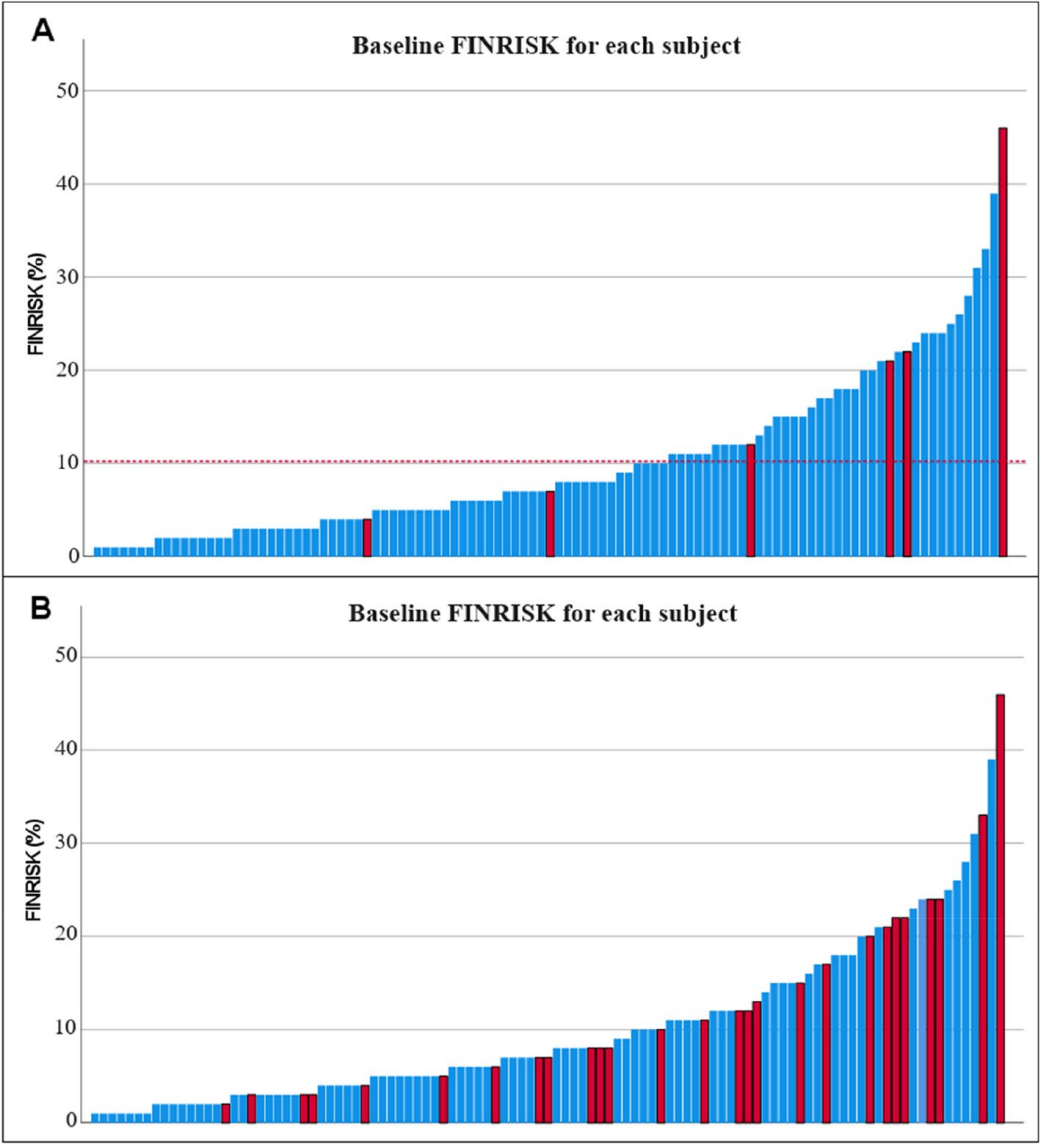
** A **The 10-year FINRISK-probability for each subject at baseline and observed strokes and myocardial infarction during 10 years. **B **The 10-year FINRISK-probability for each subject at baseline and observed composite endpoints during the follow-up of 16.4 ± 0.3 years. Red bars indicate the subjects who had either FINRISK endpoint during 10 years (**A**) or any of the composite endpoints during the whole follow-up period (**B**). In **A**, the dotted red line indicates the mean FINRISK at baseline (10.2%)

For the analysis regarding FINRISK estimation of AMI or cerebral stroke for the first 10 years we excluded two subjects for cancer related deaths occurring before the 10 years of follow-up so 103 subjects were included in the analysis. The mean FINRISK estimation of fatal or non-fatal AMI or cerebral stroke among the subjects was 10.2% (Fig. 5). The actually observed number of FINRISK endpoints during this period was six cases (5.8%): one acute myocardial infarction, four cardiovascular deaths related to myocardial infarction and one stroke. The observed FINRISK and composite endpoints according to the individual FINRISK score are presented in Fig. 6. The same exclusion of two early cancer related deaths was applied also for SCORE estimation of fatal cardiovascular events during 10 years. The mean SCORE risk for remaining 103 subjects was 3.5%, while four (3.9%) cardiovascular deaths occurred during the first 10 years.

## Discussion

The main findings of this long-term follow-up study were that Finnish men with MetS had higher incidence of CVD endpoints if their hs-CRP level was ≥ 1 mg/L than those with optimal hs-CRP concentration of < 1 mg/L. The OxLDL concentration did not seem to have any predictive effect. Subjects with normal large arterial elasticity had less CVD endpoints than subjects with borderline impaired large artery elasticity. This was not seen in subjects with different small artery elasticity. During the first 10 years, the men with MetS had fewer fatal or non-fatal AMI, cerebral stroke and fatal cardiovascular events than predicted by FINRISK.

Our results regarding the association of CVD risk and hs-CRP are in line with the increasing evidence on the interrelationship of low-grade inflammation and CVD. Accumulation of LDL-particles in the subintimal space of arterial wall is elementary process of atherosclerosis. Further, effective and prognostically favorable statin therapy has been shown to decrease concentrations of LDL-cholesterol and hs-CRP [16]. Therefore, slowing down the atherosclerotic progress or starting the healing of atherosclerosis has focused heavily in plasma cholesterol levels with good results [17]. The role of inflammation gained focus when a randomized controlled study showed that treatment of patients with established CVD and hs-CRP over 2 mg/L with monoclonal antibody canakinumab targeting interleukin-1β showed reduction in CVD events, although at cost of more fatal infections [18]. A reduction of cancer mortality in the treatment arm was also reported which is supported by the noted significance of inflammation during neoplastic progression of certain tumors [19]. Colchicine, another anti-inflammatory drug, has also been found to reduce risk of CVD events among patients with established CVD [20, 21]. There were more statin users in the low hs-CRP group (14/42 = 33%) than in the nonoptimal hs-CRP group (10/63 = 16%) (*p* = 0.049). The data on colchicine use was not collected but significant use of colchicine during the 15-year follow-up period would be unlikely.

There has been a growing interest in OxLDL as it has been thought to have a central part in the pathogenesis of atherosclerosis due to oxidative modification of macrophages and promoting dysfunction of endothelial cells [22]. The levels of OxLDL are elevated in patients with coronary, femoral and carotid artery disease [23]. OxLDL binds to several receptors including lectin-like oxidized low-density lipoprotein receptor-1 (LOX-1) enhancing endothelial activation and further oxLDL uptake in macrophages and vascular smooth muscle cells [24]. Increased levels of OxLDL together with reduction in prostacyclin production are found in hypertensive patients so they could be used to detect hypertension [25]. Our study found no difference in the prognosis of subjects with different amounts of circulating OxLDL. This could be because of the fact that all our subjects had MetS and patients with MetS are reported to have increased levels of OxLDL associated with insulin resistance and obesity [26, 27]. In our study 60/102 (59%) subjects were in th high-risk group with OxLDL ≥ 70 U/L. This could be the result of the fact that the mean systolic blood pressure of our subjects was 139 mmHg which could translate to higher amount of oxLDL and decreased prostacyclin production. It should be noted the used cutoffs for OxLDL are based on apparently healthy individuals. Our study subjects were men with MetS and 23% were using statins s the same cutoffs may not apply to them. There was no difference in the number of subjects using statins in low, moderate and high risk OxLDL groups (12/34 = 34%, 2/8 = 25%, 10/60 = 17%, *p* = NS). However, lare-scale evidence from randomized trials shows that statin therapy has significant mortality and morbidity benefit for both primary and secondary prevention from CVD. It has also been suggested that anti-inflammatory factors could downregulate LOX-1 expression [28], so further prospective large-scale studies combining inflammation and OxLDL are required.

The role of arterial elasticity in CVD progression is still uncertain and no single method has gained foothold as a reliable and practical method of arterial elasticity measurement in clinical setting. There were no subjects with clearly impaired large artery elasticity in our study even though many low-grade inflammatory diseases are associated with stiffness of large arteries [29]. The difference in CVD incidence was only reported in the groups with different large artery attributes, so the role of small artery elasticity in CVD progression could be irrelevant compared to that of the large arteries.

There was no difference in the incidence of composite endpoints in subject groups with low, medium or high FINRISK or SCORE CVD risk. We used both risk estimation systems to assess the compendium of CVD risk factors although the SCORE risk estimation system actually predicts total fatal cardiovascular risk instead of the risk for non-fatal CVD events [2]. In our study there were 17 deaths in total which equals to 16% death rate which is pretty much in line with an earlier Finnish registry-based study where overall survival for middle aged men with MetS based on NCEP classification was 90% with a slightly shorter median follow-up period of 13.7 years [1]. We included also non-cardiovascular deaths to our composite endpoint as both CVD and all-cause mortality are increased in subjects with MetS.

We found prominently less CVD endpoints at ten years than was expected by the FINRISK estimations made at the baseline. Several factors may contribute to this. The main issue could be our very small sample size. Our subjects were actively recruited to the study so they may have been more motivated to take care of their health. Our study at the baseline was not an intervention study [8]. However, participants got information concerning their individual risks found in the study. Hence, some subjects may have made substantial lifestyle changes after the results of the initial study. The progress made during last few decades in cardiovascular drug therapy and anti-tobacco legislation could have helped those not capable of sustainable lifestyle changes. On the other hand, global trends such as sedentary lifestyle and urbanization leading to increased exposure to air pollution might have caused negative effects regarding to the incidence of CVD in recent decades [30]. The FINRISK risk calculator we used is based on ten-year follow-up of three different Finnish cohorts with 9,361 men and 10,056 women in 1982–1992 [3] as our follow-up period for FINRISK-endpoints was 2004–2014. Risk models are typically based on earlier data so it is possible that the data is not fully relevant for contemporary environment and advanced therapies. The possibility of malignancies is not considered in FINRISK although some malignancies have common risk factors with CVD. The mean age of subjects at baseline of our study was 50.6 years so some malignancies and non-cardiovascular deaths were expected. Nonetheless, predicting future CVD on an individual level with risk models is challenging: a subject with a predicted 39% risk of AMI or stroke during the next 10 years had no endpoints during the whole 16-year follow-up of the study while another subject with only 4% predicted risk at baseline had AMI already at 12 months. We found the same amount of fatal cardiovascular events (4) during the first 10 years as was predicted by SCORE estimations at baseline. The amount was too low to draw any certain conclusions.

The strength of our study is the very long follow-up period of the subjects as CVD is progressive disease. We were also able to investigate several different CVD risk factors. Long-term studies regarding arterial elasticity are still sparse because of the relatively novel interest in arterial elasticity in cardiovascular research. Only four subjects were lost on follow-up. Another strength of this study is the interpretation of endpoints collected directly from the EMR instead of endpoints in registry setting collected with robust technical data extraction of International Classification of Diseases (ICD) codes prone to misinterpretation. The endpoints were real events instead of accidental findings from the increasing number of computerized tomography (CT) scans. There were no cases of periprocedural or type 2 myocardial infarctions only detected with modern day high-sensitive troponin tests and little connection to prognosis as suggested in the recent meta-analysis on nonfatal myocardial infarction as a surrogate for all-cause and cardiovascular mortality [31].

The obvious limitation of the study is the modest sample size due to the number of participants in the initial study resulting also into a limited number of events during the follow-up. Data regarding the use of colchicine and other anti-inflammatory drugs was not available, but wide-scale use during the 15-year follow-up period would be unlikely. There was missing data regarding OxLDL (three subjects) and arterial elasticity (two subjects). However, the 15-year follow-up data, hs-CRP levels, FINRISK and SCORE predictions were available for all 105 subjects. The study was also limited to a single center and geographic location so environmental confounding factors are possible. However, limiting to single center reduces possible confusion regarding locally utilized diagnostic criteria of diseases and deviations in treatment guidelines, so the registry data is uniform. All subjects were males so findings cannot be directly extrapolated to females who tend to contract CVD at slightly older age. Our biomarkers of low-grade inflammation were limited to only hs-CRP although other biomarkers such as interleukins (IL-β, 2,4,5,6, and 10) may be important as well.

## Conclusions

In conclusion, we found that during a mean follow-up of 16 years middle-aged men with MetS and hs-CRP ≥ 1 mg/L had higher risk for CVD and all-cause mortality than those with hs-CRP < 1 mg/L. This also applied to subjects with borderline decreased large artery elasticity. More research is needed to establish the role of arterial elasticity as a CVD predictor. The amount of OxLDL had no predictive value on the incidence of CVD and all-cause mortality. Men with MetS participating in the Hämeenlinna MetS Research Program without life style or drug intervention had better outcome for myocardial infarction or stroke than estimated by the FINRISK score. SCORE predicted the fatal CVD rate correctly.

## Data Availability

The datasets used and analyzed during the current study are available from the corresponding author on reasonable request.

## Abbreviations

AMI: Acute myocardial infarction
C1: Capacitive elasticity of large arteries
C2: Reflective elasticity of small arteries
CT: Computerized tomography
CVD: Cardiovascular disease
EMR: Electronic medical record
HbA1c: Glycated hemoglobin
HDL-C: High-density lipoprotein cholesterol
hs-CRP: High-sensitivity C-reactive protein
ICD: International Classification of Diseases
LDL: Low-density lipoprotein
LDL-C: Low-density lipoprotein cholesterol
LOX-1: Lectin-like oxidized low-density lipoprotein receptor-1
MetS: Metabolic syndrome
NCEP: National Cholesterol Education Program
OxLDL: Oxidized LDL
PCI: Percutaneous coronary intervention
PAD: Peripheral artery disease
SCD: Sudden cardiac death
TIA: Transient ischaemic attack
